# Long-Chain Omega-3 Oils–An Update on Sustainable Sources

**DOI:** 10.3390/nu2060572

**Published:** 2010-05-26

**Authors:** Peter D. Nichols, James Petrie, Surinder Singh

**Affiliations:** 1 CSIRO Food Futures Flagship, Division of Marine and Atmospheric Research, GPO Box 1538, Hobart, TAS 7000, Australia; 2 CSIRO Food Futures Flagship, Division of Plant Industry, PO Box 1600, Canberra, ACT 2601, Australia; Email: james.petrie@csiro.au (J.P.); surinder.singh@csiro.au (S.S)

**Keywords:** long-chain omega-3, fish oils, aquaculture, algal oils, sustainable sources, novel land plants

## Abstract

Seafood is currently the best and generally a safe source of long-chain (LC, (≥C_20_) omega-3 oils amongst the common food groups. LC omega-3 oils are also obtained in lower amounts per serve from red meat, egg and selected other foods. As global population increases the opportunities to increase seafood harvest are limited, therefore new alternate sources are required. Emerging sources include microalgae and under-utilized resources such as Southern Ocean krill. Prospects for new land plant sources of these unique and health-benefiting oils are also particularly promising, offering hope for alternate and sustainable supplies of these key oils, with resulting health, social, economic and environmental benefits.

## 1. Introduction

### 1.1. Long-Chain Omega-3 Oils–Definitions and Health Benefits

Our bodies need dietary fats and oils and, whilst some are remarkably beneficial for us [1,2], other fats can be associated with poor health. In this manuscript we concentrate on omega-3 oils (can be termed: ω3, n-3, n3; first double bond three carbons from terminal methyl end of molecule), a specific group of polyunsaturated fatty acids (PUFA, fatty acids are the main building blocks of most fats and oils). There are two different versions of omega-3 called long-chain (≥C_20_, LC) and short-chain (≤C_18_, SC). We focus here on the long-chain omega-3 (hereafter also termed LC omega-3) which include eicosapentaenoic acid (EPA, 20:5ω3), docosapentaenoic acid (DPA, 22:5ω3) and docosahexaenoic acid (DHA, 22:6ω3).

The health benefits of LC omega-3 were first reported some three decades ago. Scientists observed that Greenland Eskimos had lower amounts of heart disease than other groups despite the fact their traditional diet is actually high in fat [3]. Associated with this finding was that Eskimo blood took longer to clot. The shorter bleeding times common in Western countries can increase the risk of forming a blood clot which may trigger a heart attack. Investigations found that the traditional, seafood rich diet of the Eskimos was responsible both for the thinner blood and the healthier hearts [4]. The key ingredient of this diet was found to be LC omega-3. The human body needs fats of different types to grow and function properly and LC omega-3 oils are particularly relevant for foetal and infant growth since without LC omega-3, infant and children’s vision and growth can be impaired [5,6]. The human body cannot produce short chain omega-3 on its own, and does not convert the short chain omega-3 to the long-chain versions, especially DHA, efficiently. The issue of the poor conversion efficiency of 18:3ω3 (α-linolenic acid, ALA) to EPA and DHA in humans is also crucial to the debate on sustainable sources, with fish oil or other sources of LC omega-3 clearly the preferred and benefitting nutrient required [7,8]. When the diet does not supply sufficient omega-3, growth can be impaired and vulnerability to a number of diseases can increase. 

In addition to being required for healthy growth and development, LC omega-3 have a number of added health benefits, particularly in helping us protect against heart and blood vessel problems. In the mid 1980s CSIRO researchers discovered that LC omega-3 protect against heart attacks by maintaining normal heart rhythm [9,10]. These early observations have now been confirmed in human clinical trails. The positive impact of LC omega-3 on heart health includes: protection against heart attacks by reducing the risk of abnormal heart rhythms; maintaining healthy blood vessels; lowering of high blood pressure; influencing the narrowing of arteries; and making the blood less likely to clot. 

LC omega-3 also lower high blood fats by reducing their production in the liver and purified LC omega-3 is available as a treatment for patients with very high blood fat levels [11]. LC omega-3 oils help to combat certain inflammatory conditions such as rheumatoid arthritis [12] and it has been suggested that they may also have a positive impact on kidney function. In addition to these purely physical health benefits, it has been suggested that LC omega-3 may have benefits for neuropsychiatric disorders including depression and dementia [13,14,15]. These fats may play a role in helping with mood regulation and maintaining good cognitive function as we age although further research is needed in these areas. Here we provide an update on current and potential future sources of the LC omega-3 oils.

## 2. Current Sources of LC Omega-3

The main current source of LC omega-3 oils is seafood, including fish, crustaceans, such as lobsters, and molluscs, such as oysters and squid. Representative values for content of LC omega-3 in Australian seafood groups and individual species, together with data for other food groups, is shown in Table 1 and Table 2. Some land animals also contain LC omega-3 but generally in quite small quantities. Land plants, such as canola and linseed, contain some omega-3 although these are not the beneficial long-chain varieties. The short-chain omega-3 in land plants do not have the same health benefits as LC omega-3 [7,8]. 

In Australia, CSIRO has considerable interest in LC omega-3 and, in partnership with the Fisheries Research and Development Corporation, has profiled the LC omega-3 content of a wide range of seafood [16,17]. In terms of LC omega-3, different types of fish can have widely different amounts. For example, white-fleshed Australian wild fish, on average, contain approximately 350 mg per 150 g (representative of a typical serve size) (Table 1). However, the same quantity of slender tuna has 5,640 mg and swordfish has 1530 mg (Table 2). Different parts of the fish can contain different amounts of the LC omega-3. The season and location where the seafood was caught can also result in different contents, however, these effects are generally minor [17]. 

### 2.1. Suggested Intake of LC Omega-3

To gain the many health benefits noted above the recommended daily intake provided by many organizations for LC omega-3 can vary (see Table 3). Within Australia it is generally agreed that we need about 500 mg of the LC omega-3 oil daily, although current intake in Australia, UK and USA is estimated to be around 100–200 mg per day [18]. Two to three serves of sufficiently oily fish a week can help provide suitable intake levels. Higher intake is recommended for those with disorders that can be aided by LC omega-3 intake. The processing and cooking of seafood does not generally affect the LC omega-3 content [16,17], although many forms of cooking such as–e.g., grilling, pan-frying, baking and microwaving - are more preferable than deep frying which can add other less desirable, e.g., saturated, fats. 

### 2.2. Contaminant Concerns

While the health benefits of seafood consumption have been known for some time, there are often concerns around the potential dangers from pollutants such as mercury. These concerns can discourage people from eating fish even though mercury contamination generally applies only to large open water fish such as sharks, swordfish and large tuna. From an observational cohort study, it has been stated that advice for limiting seafood consumption during pregnancy could actually be detrimental, with it being demonstrated that risks from the loss of the LC omega-3 were greater than the risks of harm from exposure to trace contaminants in seafood [19]. Guidelines around the amounts of large fish that should be consumed are publicly available. For example, see: http://www.foodstandards.gov.au/foodmatters/mercuryinfish.cfm. These guidelines give special emphasis for people who may be more at risk from mercury, such as pregnant women. However, the warnings around excessive consumption of large fish have at times been seen as a warning regarding all fish. The mercury issue has also been overstated, as seafood contains selenium which counteracts the effects of mercury [19]. The guidelines around the consumption of large fish such as shark and swordfish need to be taken in account, although it is noted that mercury is not a problem for all fish and the health benefits from consuming fish and the LC omega-3 it contains far outweigh the risks [19].

**Table 1:** Average LC omega-3 content of wild and farmed Australian seafood together with other food groups ^1^.

Food	mg/150 g (wet weight ^2^)
**Wild Australian seafood**	
Fish	350
Shellfish	225
Prawns	180
Lobster	160
**Farmed Australian fish**	
Striped perch	3,700
Atlantic salmon	2,985
Barramundi	2,960
Silver perch	1,200
**Other food groups**	
Turkey	40
Beef	40
Chicken	40
Pork	40
Lamb	30

^1^ Data from [16,17], includes EPA, DPA and DHA

^2^ 150 g is representative of a typical serve size.

**Table 2:** Wild-caught Australian seafood containing more than 450 mg/150 g (raw) of LC omega-3, data derived from [16,17].

Marketing Name	Scientific Name	Oil (g/100g)	Total LC omega-3 ^1^ (mg/150 g)
Slender tuna	*Allothunnus fallai*	16.5	5,640
Swordfish	*Xiphias gladius*	7.7	1,530
Escolar ^2^	*Ruvettus pretiosus*	17.8	1,530
Banded morwong	*Cheilodactylus spectablis*	3.2	1,230
Alfonsino	*Beryx splendens*	5.2	1,195
Whitebait	*Lovettia sealii*	2.6	1,100
Escolar ^2^	*Lepidocybium flavobrunneum*	19.2	1,075
Big-eye trevally	*Caranx sexfasciatus*	4.7	1,065
Whitebait	*Galaxias maculatus*	3.3	1,030
Blue mackerel	*Scomber australasicus*	3.8	760
Australian bonito	*Sarda australis*	1.5	650
Gemfish	*Rexea solandri*	2.6	640
Rudderfish	*Centrolophus niger*	14.4	620
Spanish mackerel	*Scomberomorus commerson*	3	575
Sweep	*Scorpis lineolatus*	1.3	555
Australian herring	*Arripis georgianus*	1.7	540
Western blue grouper	*Achoerodus gouldii*	3.6	540
Bigspine boarfish	*Pentaceros decacanthus*	1.5	530
Eastern Australian salmon	*Arripis trutta*	1.1	505
Spotted mackerel	*Scomberomorus munroi*	1.2	500
School mackerel	*Scomberomorus queenslandicus*	1.1	490
Grey mackerel	*Scomberomorus semifasciatus*	1.1	490
Tailor	*Pomatomus saltatrix*	1.3	490
Threadfin emperor	*Lethrinus genivittatus*	2.6	490
Bight redfish	*Centroberyx gerrardi*	0.5	485
Pilchard	*Sardinops neopilchardus*	1.2	470
Blue eye trevalla	*Hyperoglyphe antarctica*	1.3	470

^1^ includes EPA, DPA and DHA

^2^ consumption of escolar may cause illness

### 2.3. Sustainability of Current Sources of LC Omega-3

Against the increasingly recognized literature and other public materials available on the health benefits of LC omega-3 oils, the global decline in wild-harvest fish stocks is also a more recently recognized issue. In a widely-cited study [15], it was estimated that the amount of large fish in the oceans is only 10% of pre-industrial times, although this has been disputed [16]. It has been further stated that ocean and fish stock health are considered so under threat that by 2048 ‘all commercial fish and seafood may collapse’ [17]. This view on the state of the world’s fisheries is not unanimous with recent progress establishing some common ground between the sometimes opposing views. Progress is also being made by many countries towards rebuilding global fisheries and sustainability and different approaches are required for industrial fisheries compared to small-scale fisheries [18]. An interesting aspect in the equation is that the environmental impact of marine fisheries is seen as much less than for production of animal protein from agriculture that requires the removal of forest and in many dimensions is also less than for vegetarian diets. (Ray Hilborn, personal communication).

Australian research has also shown that stocks of up to 60 species e.g., eastern gemfish, snapper in NSW and WA, are under pressure from environmental damage and over fishing [19], although in general Australian marine resources are regarded as well managed [20]. These declines, when coupled with the ongoing growth of world aquaculture, increasing world population coupled with increased seafood consumption in western diets, means that new sources of LC omega-3 oil are required.

Several calculations have been recently performed to examine global consumer needs for the health-benefiting LC omega-3 oils [21]. Using - a daily 500 mg requirement as is recommended by many bodies, a global population reaching 8 billion by 2025 (United Nations estimate in 2007), and assuming fish contain 2 to 5 mg/100 g oil, it was estimated that the current global fish harvest (93 Mt per annum) will not supply this requirement. A second calculation performed for table fish, assuming an average LC omega-3 content of 450 mg/150 g flesh (average serve size), a harvest of 70 Mt, and 50% edible portion. Neither calculation takes into account processing inefficiencies, which therefore further emphasizes a predicted shortfall in fisheries catch meeting global needs for the unique and health benefiting LC omega-3 oils.

**Table 3:** Selected suggested LC omega-3 (EPA + DHA) intakes for adults available from various agencies and bodies.

Authority / Group	mg/day
Omega Workshop, Adelaide, Australia, 2002 [20]	300–400
SACN/COT, UK 2004 [21]	450
National Heart Foundation, 2008 [22]	500
American Dietetic Association and Dietitians of Canada, 2007 [23]	500
FAO/WHO Expert Consultation, 2008 [24]	250–2,000*
American Heart Association, 2002 [25]	
Coronary Heart Disease sufferers	1,000
Those seeking to reduce triacylglycerols (blood fats)	2,000–4,000
Australia and New Zealand (suggested dietary targets), 2006 [26]	
Female	430
Male	610

* for secondary prevention of coronary heart disease

## 3. Fish farming: Current Status and Looking to the Future

Over the past two decades the farming of fish has increased considerably. The amount of farmed fish on the market will soon surpass that of wild caught fish [37]. From an environmental point of view this seems very positive as the pressures on wild fish stocks can be quite severe as noted above. Beyond concerns for the environment it is also becoming increasingly clear that global fish stocks will not be able to meet human’s nutritional needs in the future [36]. Hence, farming fish appears to be a good opportunity to increase both global production and consumer needs. 

As aquaculture expanded in the past decades, most carnivorous and many omnivorous farmed fish were initially all fed other fish (e.g., tuna ranching) or fish-derived products in pellet form generally comprising fish meal and fish oil as the major ingredients (e.g., salmon farming). Wild fish stocks were being harvested and used to feed the farmed fish. Due to increasing global demand for fish oil and environmental issues more and more suppliers are starting to use plant-based and other sources of ingredients and oils as feed for their fish [35,37,38]. However, the problem for consumers is that farmed fish do not naturally produce large quantities of LC omega-3, since in the wild fish gain their LC omega-3 from small aquatic plants, microalgae, that are consumed at the bottom of the marine food chain. The LC omega-3 oil is then passed up the food chain and stored in the fish bodies. As land plants produce the far less useful short chain (C_18_) omega-3, feeding fish on land plant derived oils containing the short chain ALA results in far lower accumulation of LC omega-3 in the farmed fish and consumers do not gain the same health benefits.

In contrast, farmed fish can have even higher concentrations of LC omega-3 than fish caught in the wild when fed on fish oil based diets. For instance, the main farmed fish in Australia, such as Tasmanian Atlantic salmon from southern waters and barramundi from northern waters, provide up to 10 times more LC omega-3 (~3,000 mg/150g) than the average wild fish (Table 1). Until recent years Australian and New Zealand fish farms were largely using fish products in feed so the LC omega-3 content remained high. Further pressures on wild fish stocks will likely result in increased use of oils derived from land plants and or other ingredients in feeds, with: (i) lower relative levels (as % of total fatty acids), (ii) lower absolute content of the LC omega-3 oil and (iii) lower ω3 to ω6 ratio resulting than has previously occurred in farmed fish [34]. As the relative levels of these key LC omega-3 oils decrease, linoleic acid (LA, 18:2ω6) and oleic acid (OA, 18:1ω9c) increase; OA and LA are derived from the non-marine ingredients that are being increasingly substituted into aquafeeds [35]. However, farmed seafood species generally still remain an excellent source of the LC omega-3 oils. In Australia for example, farmed Tasmanian Atlantic salmon is widely available and remains an excellent source of LC omega-3 oil (1,610 mg/150 g serve, unpublished data 2010), clearly one of the best of all foods available for consumers.

A number of avenues can be considered for turning the current trend in LC omega-3 oils in farmed fish around. Practices at fish farms could be examined to make better use of the wild fish stocks, and also using ‘finishing diets’; the latter involves feeding fish on diets comprising plant or other ingredients during the grow-out phase, followed by the use of a fish oil diet during the final finishing period prior to harvest [37]. Making better use of current wild harvest ‘waste’ products, such as the liver and head, as sources of LC omega-3 oils, also could be expanded. The range of fish used could be widened, including species such as the underutilized Antarctic krill. Krill oil has attracted increasing interest in recent years and contains high (~30%) relative levels of EPA and DHA together with the carotenoid pigment astaxanthin, with much of the LC omega-3 present in phospholipids rather than the conventional triacylglycerol form that occurs in fish oil [34]. Considerable caution would be needed for increasing the harvest of this species as it underpins the Southern Ocean food web. The problem still remains of decreasing wild fish stocks and the need for farmed fish to also have high amounts of the LC omega-3. 

## 4. New Alternate Sources of LC Omega-3

A number of options are currently being explored to assist with potential future problems of decreased populations of wild fish and the health and nutritional needs of consumers. Sources of LC omega-3 are currently being developed from microalgae by several companies [35,39,40], with considerable interest in single cell organisms (SCO) such as heterotrophic dinoflagellates and thraustochytrids and some species from other algal groups. Thraustochyrids were originally thought to be closely related to primitive fungi, although have more recently been assigned to the subclass Thraustochytridea (Chromista, Heterokonta), aligning them with heterokont algae such as diatoms and brown algae [41]. Whilst SCO sources are finding increasing use in infant formulas and other niche areas including nutrition of fish larvae, it is presently generally considered too expensive for use in fish farming, which is the current main user (~90% of global production) of fish oil containing LC omega-3 oils. As more research is conducted, including in the growing field of biofuels, it may be possible to reduce production costs for microalgae. The genetic modification of microorganisms such as yeast to produce LC omega-3 is also a future possibility. Yeast and similar bioproducts may be targeted as they can easily be fermented on a large scale, potentially making the production of LC omega-3 oil relatively efficient [42].

In an alternative approach to the use of microorganisms, a number of international teams are now using genetic engineering technology to allow land plant crops to produce the health-benefitting LC omega-3 oils (see Table 4); several reviews are available and provide summaries of the considerable progress to date towards this goal [43,44]. This alternative approach to obtaining LC omega-3 oils has resulted from concerns on over-fishing and about pollution of the marine environment [35,36,44], in addition to the increasing recognition of the enormous health and socio-economic benefits of these oils and the expanding global population and finally the clear need for new sustainable sources. 

There are two distinct pathways leading to the biosynthesis of LC-PUFA in nature: 1. The conventional (aerobic) fatty acid desaturation/elongation pathway, and 2. The anaerobic polyketide synthase (PKS) pathway. The PKS route is similar to those involving the PKS complexes in antibiotic synthesis. No reports of successful expression of the PKS pathway in land plants have emerged to date. In contrast, insertion of the aerobic pathway leading to DHA production, into omega-3 C_18_ PUFA accumulating land plants, has been reported in a number of studies. Transfer of genes from microorganisms to land plants led to accumulation of 2.4% EPA and 0.5% DHA in *Arabidopsis* (Mouseear cress, later improved to 4% EPA and 1% DHA) [45]–(the first reporting of a (model) land plant with DHA), 15% EPA and 0.2% DHA in *Brassica juncea* (Indian mustard) [46], 19.6% EPA and 3.3% DHA in soybean (Table 4) [47]. 

These initial findings from a number of research teams clearly indicate the feasibility of developing grain crops with significant amounts of LC omega-3 oil. Further research is required to develop commercial oilseed crops with LC omega-3 oil and increased levels of DHA in particular are still required. It is interesting to point out, that as little as one tablespoon (~10 mL) of soybean oil containing 20% of EPA could make a significant contribution to the recommended dietary intake of the beneficial omega-3 LC-PUFA. Considerable effort is now being focused on increasing the levels of DHA and it is not unrealistic to hope that DHA levels of 20% can be achieved in transgenic plants in the next few years. 

Future novel land-based plants will likely provide the most economically viable source LC omega-3 oil for aquaculture and other applications. A land plant source of LC omega-3, if achieved and assuming their cultivation will be permitted, will be cheaper than using yeast or microalgae and could be used in fish feeds and other feed and food products to help deliver increased human health benefits [35,42]. Research involving the use of microbial genes in land plants has so far led to increases the production of LC omega-3 in a number of land plant species (Table 4). This future may be as little as five years away [48], although perhaps longer in the context of aquaculture feeds and for products for direct human consumption in particular to meet all the permitting requirements that are likely to be in place in 15 years [35,42].

**Figure 1:**
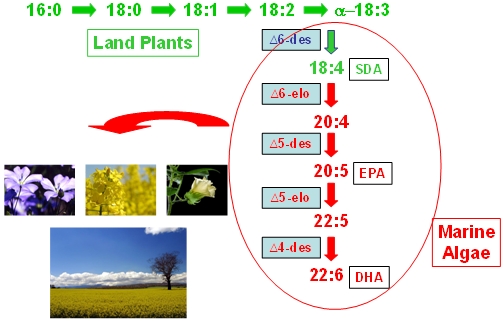
Schematic representation of fatty acid pathway for new land plants containing EPA and DHA, with incorporation on selected microalgae-derived genes. Although the Δ6 route is shown here, another pathway for LC omega-3 biosynthesis (Δ9, from ALA) also exists.

**Table 4:** Comparison of fatty acid composition (expressed as g/100 g of total fatty acids) for new land plants containing the C_18_ omega-3 PUFA stearidonic acid, arachidonic acid or LC omega-3 oils.

	Ref	SDA	EPA	ARA	DHA
CSIRO: oilseeds (includes model plants)	[49]	10			
	[45]		5		1
	[49]	1	26	2	
	[50]			22	
BASF: mustard	[46]		15	7	1.5
Monsanto: soya bean	[51]	20			
Dupont: soya bean	[47]		20		3
Farmed salmon					
*Fed fish oil diet*	[16,17]		10		17
*Fed plant oil diet*	[52]		2.2		5

Abbreviations: Abbreviations: SDA, stearidonic acid EPA; EPA, eicosapentaenoic acid; ARA, arachidonic acid; DHA, docosahexaenoic acid.

Research in the area of land plant sources of LC omega-3 has the potential for significant health, social, environmental and commercial benefits. Consumer and industry acceptance of this biotechnology and new health and safety requirements set by regulatory bodies will be needed for oils from these novel land plants before they can be used by the aquaculture and other industries. Recentassessments of perceived consumer acceptance of novel land plant LC omega-3 technologies in Australia and the USA have reported that farmed fish were preferred as a delivery mechanism over capsules or functional foods [53,54]. It is likely that consumers will accept oil from a GM crop as an ingredient of aquafeeds as demand for fish oil intensifies, as knowledge about fishing impacts and benefits to human health of LC omega-3 increases, and as farmed fish prices decrease owing to reduced ingredient costs [35]. 

Considerable effort is now being focused on increasing the levels of LC omega-3 in land plants and it is not unrealistic to foresee that sufficiently high concentrations can be reached in the next few years. Similarly, research is needed to open up new avenues for delivery of LC omega-3 oils to consumers via grains including e.g. whole grains in foods, vegetative plant tissues, extracted oil in spreads, extracted oils as food ingredients and aquaculture and livestock feeds.

## 5. Conclusion

LC omega-3 oils are required for ongoing health and vitality and to provide consumers with benefits across a range of areas, including heart disease, stroke, rheumatoid arthritis, some forms of cancer and other disorders. It is recognized that an increase in LC omega-3 consumption is required, which, currently, means at least two serves of appropriately oily fish per week. Future supplies of the beneficial LC omega-3 oils may not be sufficient for the predicted increasing demands for their inclusion in both feeds and food and nutraceutical products. In addition, with the wild harvest fisheries from which the LC omega-3 are obtained under threat, we need to explore alternate means to ensure their future supply while still protecting the environment and ocean life. Potential alternate sources of LC omega-3 for use in aquaculture and other application areas include single cell microalgal oils and/or biomass, and under-utilized marine sources including krill, although expanding the fishing of these building blocks of the marine ecosystem should be carefully considered before a marked increase in exploitation of krill resources occurs. Finally, the exciting prospect is evolving for the further development and application of novel transgenic oilseeds with several research groups active in this area.
